# Chagas Disease Vector Control in a Hyperendemic Setting: The First 11 Years of Intervention in Cochabamba, Bolivia

**DOI:** 10.1371/journal.pntd.0002782

**Published:** 2014-04-03

**Authors:** Natalisisy Espinoza, Rafael Borrás, Fernando Abad-Franch

**Affiliations:** 1 Departamento de Microbiología y Ecología, Facultad de Medicina y Odontología, Universitat de València, Valencia, Spain; 2 Instituto Leônidas e Maria Deane – Fiocruz Amazônia, Manaus, Brazil; Universidad Autónoma de Yucatán, Mexico

## Abstract

**Background:**

Chagas disease has historically been hyperendemic in the Bolivian Department of Cochabamba. In the early 2000s, an extensive vector control program was implemented; 1.34 million dwelling inspections were conducted to ascertain infestation (2000–2001/2003–2011), with blanket insecticide spraying in 2003–2005 and subsequent survey-spraying cycles targeting residual infestation foci. Here, we assess the effects of this program on dwelling infestation rates (DIRs).

**Methodology/Principal Findings:**

Program records were used to calculate annual, municipality-level aggregate DIRs (39 municipalities); very high values in 2000–2001 (median: 0.77–0.69) dropped to ∼0.03 from 2004 on. A linear mixed model (with municipality as a random factor) suggested that infestation odds decreased, on average, by ∼28% (95% confidence interval [CI_95_] 6–44%) with each 10-fold increase in control effort. A second, better-fitting mixed model including year as an ordinal predictor disclosed large DIR reductions in 2001–2003 (odds ratio [OR] 0.11, CI_95_ 0.06–0.19) and 2003–2004 (OR 0.22, CI_95_ 0.14–0.34). Except for a moderate decrease in 2005–2006, no significant changes were detected afterwards. In both models, municipality-level DIRs correlated positively with previous-year DIRs and with the extent of municipal territory originally covered by montane dry forests.

**Conclusions/Significance:**

Insecticide-spraying campaigns had very strong, long-lasting effects on DIRs in Cochabamba. However, post-intervention surveys consistently detected infestation in ∼3% of dwellings, underscoring the need for continuous surveillance; higher DIRs were recorded in the capital city and, more generally, in municipalities dominated by montane dry forest – an eco-region where wild *Triatoma infestans* are widespread. Traditional strategies combining insecticide spraying and longitudinal surveillance are thus confirmed as very effective means for area-wide Chagas disease vector control; they will be particularly beneficial in highly-endemic settings, but should also be implemented or maintained in other parts of Latin America where domestic infestation by triatomines is still commonplace.

## Author Summary

Chagas disease is among the most serious public health problems in Latin America; the highest prevalence of infection by its causative agent, the parasite *Trypanosoma cruzi*, has historically been recorded in some parts of Bolivia. In the early 2000s, a massive insecticide-spraying program was set up to control dwelling infestation by the blood-sucking bugs that transmit the disease. Here we provide a detailed assessment of the effects of this program in the Department of Cochabamba, one of the most highly-endemic settings worldwide. Our analyses show that municipality-level dwelling infestation rates plummeted from over 70–80% in 2001–2003 to about 2–3% in 2004–2011. This residual infestation was higher in the capital city and, more generally, in municipalities where montane dry forests dominate – probably because wild populations of the main vector, *Triatoma infestans*, are common in that eco-region. Despite the impressive early achievements of the program, with about 0.5 million people protected from contagion, sustained disease control will require fully operational long-term surveillance systems.

## Introduction

Chagas disease, caused by infection with the parasite *Trypanosoma cruzi*, is among the most serious public health problems in Bolivia [1]–[9]. In particular, the disease has historically been hyperendemic in some areas of the country in which *Triatoma infestans*, the main vector of human Chagas disease, is frequently found infesting houses. The Department of Cochabamba is one such area [5], [7]–[9].

Domestic *T. infestans* populations were accidentally introduced in most of their past range across South America, allowing for their elimination through area-wide control campaigns based on the spraying of houses and peridomestic structures with residual insecticides [10]. However, it has become progressively clear that wild populations of this highly efficient vector species are widespread both in the inter-Andean valleys of central- and south-eastern Bolivia, including Cochabamba, and across the semi-arid Gran Chaco [11]–[21]. These wild populations may act as the sources of re-infesting vectors in their natural ranges, and this might hamper long-term efforts to keep dwellings vector-free [10], [22].

Based on the decades-long, successful experience of domestic Chagas disease vector control through pyrethroid insecticide spraying in the Southern Cone countries of South America (e.g., [1], [5]) and elsewhere (e.g., [22]–[25]), Bolivia launched an ambitious Chagas Disease Control Program (CDCP) in the early 2000s [1], [26], [27]. Here we assess the long-term effects of the CDCP on the frequency of dwelling infestation by triatomine bugs (primarily *T. infestans*) in the Department of Cochabamba. Specifically, we aimed at quantifying how dwelling infestation rates varied with increasing control effort as well as from one year to the next over an 11-year period including a pre-intervention phase and a seven-year follow-up phase. Additionally, we asked whether the widespread occurrence of wild *T. infestans* foci in the region, and particularly in certain eco-regions, could compromise vector control efforts to any serious degree, thus gauging the need for continuous entomological surveillance [16], [22], [28].

## Materials and Methods

### Ethics statement

N.E. obtained written permission to use CDCP data from the head of the Epidemiology Unit of the Cochabamba Department Health Service (document CITE/SEDES/ACE/016/09). All data on dwellings and individuals were anonymized.

### Setting

Cochabamba is one of the nine political Departments of Bolivia; 2010 demographic estimates indicate that about 1.9 million people live in the Department, ∼35–40% of them in rural localities; the municipality of Cercado, which includes the capital city, Cochabamba, has ∼620,000 inhabitants (Instituto Nacional de Estadística de Bolivia, INE [www.ine.gob.bo]). Poverty affects ∼35% of the population [29]; hence, official census data indicate that, in 2001, nearly 35% of houses had earthen floors and only about 40% had brick/cement walls (INE). In Bolivia as a whole, ∼45% of dwellings are still substandard (∼72% for the lowest-income quintile of the population; see http://sedlac.econo.unlp.edu.ar/eng/statistics-detalle.php?idE=39), and low-quality housing is known to favor infestation by triatomine bugs [30]. In Cochabamba, *T. infestans* is highly dominant, but *T. sordida* may also infest houses and bugs identified as *T. guasayana* or *Panstrongylus megistus* are sporadically collected (see below and refs. [30], [31]). As for other parts of Bolivia [1]–[4], [6], the prevalence of human infection by *T. cruzi* in Cochabamba used to be among the highest worldwide, with published reports suggesting mean values ∼20% – but reaching up to ∼70% or more among adults in some communities [7]–[9], [31], [32]. Dwelling infestation by *T. infestans* is the key determinant of this epidemiological scenario, with *T. sordida* probably playing no significant role in transmission [33]. Therefore, vector control aimed at domestic *T. infestans* populations is a crucial component of the Bolivian CDCP [1], [10], [26], [27], [31].

**Figure 1:**
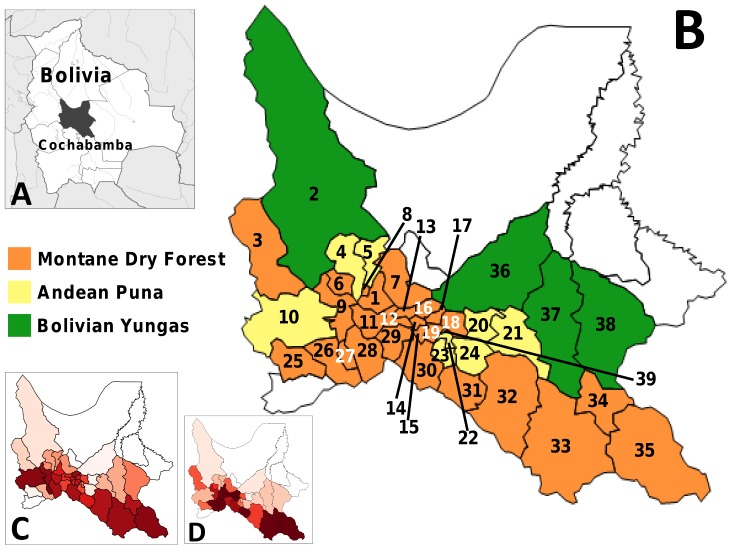
The Department of Cochabamba, Bolivia. **A**, location of the Department of Cochabamba in Bolivia. **B**, Municipalities included in the Chagas Disease Control Program (CDCP), and therefore in our assessment, are color-coded according to the predominant (≥50% of territory) eco-region. Blank municipalities were not considered at risk by the CDCP and were not included in the analyses. 1, Cercado (includes the capital city, Cochabamba); 2, Morochata; 3, Independencia; 4, Quillacollo; 5, Tiquipaya; 6, Vinto; 7, Sacaba; 8, Colcapirhua; 9, Sipe Sipe; 10, Tapacarí; 11, Santiváñez; 12, Arbieto; 13, Tolata; 14, Cliza; 15, Toco; 16, San Benito; 17, Punata; 18, Arani; 19, Villa Rivero; 20, Vacas; 21, Pocona; 22, Cuchumuela; 23, Sacabamba; 24, Alalay; 25, Tacopaya; 26, Arque; 27, Sicaya; 28, Capinota; 29, Tarata; 30, Anzaldo; 31, Vila Vila; 32, Mizque; 33, Aiquile; 34, Omereque; 35, Pasorapa; 36, Tiraque; 37, Totora; 38, Pojo; 39, Tacachi. **C**, maximum dwelling infestation rates recorded in each municipality over the study period, illustrating overall among-municipality variation in baseline risk. **D**, percent of municipal territory originally covered by montane dry forest (disregarding deforestation or other land-use changes). In **C** and **D**, the color scale goes from 0% (paler/pink) to 100% (darker/red) as in Figure 2.

Our analyses cover 39 of the 45 municipalities of Cochabamba (Figure 1). These municipalities lie within the ‘at-risk area’ specifically targeted by the CDCP; for each of them, at least 5 years of infestation survey data were available for assessing intervention results over the period of interest (2000–2011; see Table S1). The study municipalities are mostly located on the southern and western (Andean) parts of the Department, spanning three major eco-regions (*sensu* Olson et al. [34]; Figure 1), and include the temperate, montane dry forest valleys where wild *T. infestans* foci are widespread [16], [18].

### Intervention

Insecticide spraying was the central tactic of the Cochabamba CDCP. Synthetic pyrethroids (mainly alpha-cypermethrin 20%, 25 mg a.i./m^2^) were applied by trained CDCP staff in all dwellings of at-risk localities following standard procedures [35]; in Cochabamba, 205 localities were considered at high, 647 at moderate, and 2024 at low risk within the 39 at-risk municipalities (CDCP data). The intervention proceeded in three main phases, with logistic constraints resulting in some variation in the timing and coverage of control actions across municipalities. Briefly, baseline infestation surveys (mainly 1999–2001) were followed by blanket insecticide spraying over two or three rounds (mainly during 2001–2005); finally, infestation surveys and spraying were targeted at dwellings reporting residual/re-emerging infestation foci, whether by dweller notification or by active bug searches by CDCP staff. Bug searches and spraying were scheduled at different times depending on the implementation and results of previous phases [35]. Due to financial constraints, the CDCP did not conduct any activities in 2002. Overall, a median ∼62% of target houses (i.e., those in at-risk municipalities) were searched for bugs each year (inter-quartile range 17–100%); much lower values in Cercado (median 2.7%, inter-quartile range 0.99–9.5%) likely reflect the fact that only some periurban neighborhoods were considered at risk within the capital city, although an estimate of 59.0% and 65.3% of target houses were investigated in 2003 and 2004, respectively (Table S1).

### The data

The Cochabamba CDCP provided municipality-level data on dwelling infestation (numbers of dwellings surveyed and found infested) and control activities (houses sprayed and amount of insecticide used) for each year (see Table 1), as well as on triatomine catches (2007–2010; Table S2). Demographic and social-economic data were retrieved from the Bolivian INE (www.ine.gob.bo) and the United Nations Development Program [29]. Eco-region data (Table 2, Figure 1) were derived from digital maps available from the World Wildlife Fund (http://worldwildlife.org).

**Table 1:** Dwelling infestation by triatomines and vector control effort across 39 municipalities in Chagas disease risk areas, Cochabamba, Bolivia, 2000–2011*.

Year	Dwellings			Insecticide•		Municipalities
	Surveyed	Infested#	Infested (SEM)§	Mean (SEM)	Median (IQR)	*N*
2000	28,721	0.75	0.77 (0.03)	10.74 (3.23)	0.00 (0.00–16.23)	12
2001	43,998	0.68	0.70 (0.04)	19.58 (3.70)	1.26 (0.00–40.64)	20
2003	236,946	0.26	0.30 (0.04)	30.64 (2.94)	31.87 (10.20–46.90)	38
2004	228,902	0.02	0.04 (0.01)	12.94 (2.09)	8.12 (2.21–21.85)	36
2005	188,777	0.03	0.04 (0.01)	14.87 (3.16)	5.70 (2.09–26.36)	39
2006	113,091	0.02	0.02 (0.00)	9.25 (2.99)	1.66 (0.44–8.29)	38
2007	64,562	0.04	0.04 (0.01)	2.94 (0.83)	0.71 (0.28–2.85)	37
2008	107,866	0.03	0.03 (0.01)	2.26 (0.43)	0.86 (0.38–3.28)	39
2009	114,382	0.03	0.02 (0.01)	3.82 (1.44)	0.82 (0.33–3.91)	39
2010	117,545	0.03	0.03 (0.01)	0.44 (0.18)	0.12 (0.02–0.48)	37
2011	96,170	0.03	0.03 (0.01)	3.30 (1.11)	0.68 (0.15–1.98)	36

*No data available for 2002.

• Amount of insecticide (in cc) used per census inhabitant (municipality-level summary measures).

# Observed overall proportion of infested dwellings.

§ Mean municipality-level proportion of infested dwellings.

SEM, standard error of the mean; IQR, inter-quartile range; *N*, number of municipalities with data.

**Table 2:** Dwelling infestation by triatomine bugs in 39 municipalities within Chagas disease risk areas, Cochabamba, Bolivia, 2000–2011*: eco-regional descriptive statistics.

Eco-region	Municipalities•	Proportion of dwellings found infested#				
		Mean	SEM	Median	IQR	Maximum
Dry forest§	27	0.14	0.015	0.03	0.01–0.84	0.96
Puna	8	0.07	0.020	0.01	0.00–0.02	0.93
Yungas	4	0.04	0.016	0.01	0.00–0.03	0.45
Overall	39	0.12	0.012	0.02	0.01–0.07	0.96

*No data available for 2002.

• Number of municipalities with ≥50% of territory originally covered by each eco-region type (i.e., disregarding deforestation or other land-use changes).

# Summary measures of municipality-level infestation rates across years over the study period.

§ Non-parametric rank-sum tests and Welch Anova (allowing for unequal variances) suggested higher overall infestation rates in these municipalities.

SEM, standard error of the mean; IQR, inter-quartile range.

### Data analyses

#### Descriptive statistics

The first phase of the analyses focused on summarizing major data features in tables and graphs. These included (i) geographic, ecological and demographic characteristics of the study area and municipalities; (ii) descriptors of the vector-control intervention; and (iii) the observed time-trends of house infestation by Chagas disease vectors in the study area and municipalities. For these analyses, we calculated measures of central tendency (mean, median) and dispersion (standard errors [SE], standard deviations [SD], quantiles); 95% confidence intervals (CI_95_) of simple proportions were estimated using the Agresti-Coull method [36]. Microsoft Excel spreadsheets (Microsoft Corp., Redmond, WA, USA) and JMP 9.0 (SAS Institute, Cary, NC, USA) were used for these descriptive analyses.

#### Modeling

In a second phase, we used linear mixed models to derive statistical estimates of intervention effects and year-to-year infestation rate changes while taking into account both the repeated-measures structure of the dataset and the effects of potential confounders. Models were fit via restricted maximum likelihood (REML) [37] as implemented in JMP 9.0. Following Warton and Hui [38], the response variable (proportion of infested dwellings in each municipality and year) was logit-transformed to approximately satisfy linear modeling assumptions; the smallest non-zero infestation value recorded in the entire dataset (0.000288) was added to the numerator and denominator of the transformation formula to avoid undefined values when observed infestation was zero [38]. We used diagnostic plots (residual versus fitted plots, residual frequency distributions, and normal quantile plots) to check that basic modeling assumptions were reasonably met [38].

We first evaluated a model in which intervention effort was measured as the amount of insecticide used per census inhabitant in each municipality during the previous year (log_10_-transformed, in cc). The structure of the model reflects our aim of assessing average intervention effects on dwelling infestation rates at the municipality level while (a) accounting for the likely non-independence of repeated municipality surveys (by declaring municipality as a random factor) and (b) controlling for the possible effects of the following covariates/confounders (specified as fixed effects):

- Temporal autocorrelation, represented by a covariate specifying, for each municipality and year, infestation rates ascertained the previous year; this continuous covariate reflects our belief that, for any given municipality, infestation in year *t* would likely depend on infestation in year *t*–1;
- Eco-regional variation among municipalities (see Figure 1 and Table 2), which might influence the overall, ‘baseline’ probability of triatomine occurrence. After preliminary analyses, eco-regional variation was measured as the (log_10_-transformed) percent of municipal territory that originally corresponded to montane dry forest, with values calculated in the GRASS GIS environment (http://grass.osgeo.org/);
- The Human Development Index (HDI), an average, composite measure of social and economic development [39] for each municipality. Since municipality-level HDI values were available only for 2001 and 2005 and remained largely stable over that period (mean difference 0.002, range −0.007 to 0.05), we used 2005 values as provided by the INE (mean and median ∼0.55, variance 0.013, inter-quartile range 0.45–0.63).

A second mixed model was used to quantitatively assess year-to-year changes in municipality-level dwelling infestation odds; for this, the ‘intervention effort’ predictor was replaced by a ‘year’ ordinal predictor, with the rest of model structure specified as above. We compared model performance using AICc (second-order Akaike information criterion) and BIC (Bayesian information criterion) scores [40], [41]. In both models, the outcome variable was weighted by the proportion of target houses surveyed in each municipality and year (Table S1) to account for unequal bug-search effort.

**Figure 2:**
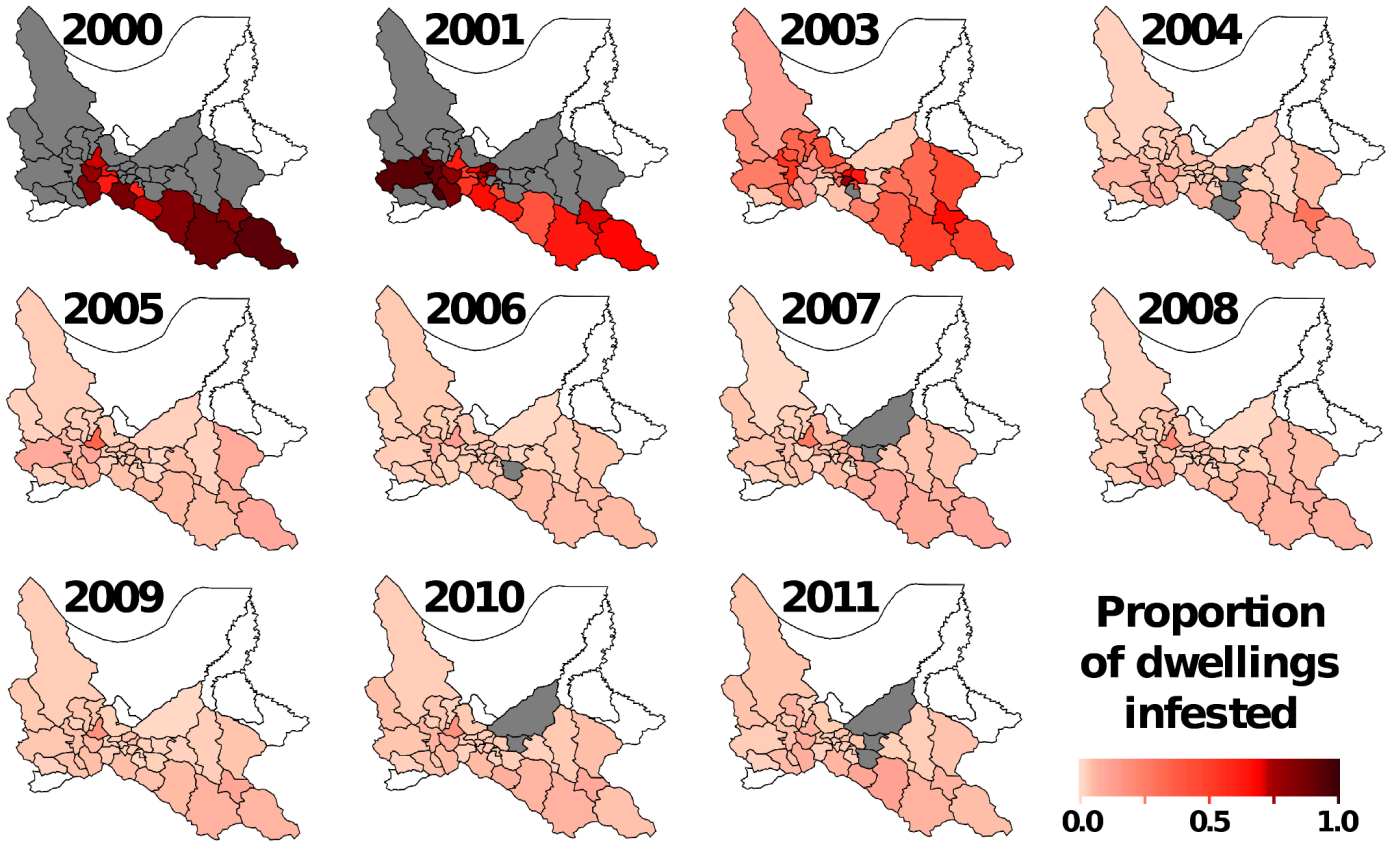
Dwelling infestation by triatomine bugs in at-risk municipalities, Cochabamba, Bolivia. Choropleth maps showing observed dwelling infestation values in each municipality and year. Color codes: pale-pink-to-dark red, proportion of infested dwellings (see scale bar); blank, municipalities not considered at risk and not included in the vector control program nor in the analyses; grey, municipalities without year-specific data.

Not all municipalities had infestation data for each year within the study period (see Figure 2 and Table S1); missing municipality-years were excluded from the analyses. We again note that the CDCP did not conduct any activities in 2002; this year was therefore ignored in all our analyses. Note also that, since our models include one-year-lagged terms, no estimates are derived for the year 2000.

## Results

### Descriptive results

The initial surveys revealed extremely high infestation rates, with mean municipality-level values above 70% of investigated dwellings (Table 1, Figure 2). The extreme case was Sicaya, where 539 out of 562 dwellings surveyed in 2001 (i.e., 95.9%) were infested; four further municipalities had infestation rates above 90%, with a total of 4436 dwellings infested out of 4842 investigated. Average infestation rates began to decline by 2003, when the effects of the CDCP were becoming evident in some municipalities: two of them still recorded infestation rates >80%, while seven were already below 3% (Figures 2 and S1). In contrast, dwelling infestation rates were overall strikingly reduced by 2004, with median values about one order of magnitude lower than those recorded in 2000–2001 (Table 1, Figure S1). With the exception of a few municipalities, such low values were sustained over the rest of the assessment period (Figure 2) with a relatively modest investment in insecticides, particularly from 2007 on (Table 1).

It is however important to note that observed dwelling infestation rarely reached zero in any particular municipality and year; when none of the surveyed dwellings were found to be infested in a given municipality, this was seldom consistent across different annual assessments (Table S3). In most cases, annual mean municipality-level infestation values remained fairly constantly at about 2–4% after 2004 (Table 1). In Cercado (which includes the capital city, Cochabamba), dwelling infestation rates were in the range of 11–33% during 2003–2010, with the exceptions of 2004, with a reported rate of 1.45% potentially due to a typing error, and 2011, when a 6.95% rate was reported.

Figures 1C and 2 illustrate the spatial patterns of infestation across the assessment period; they indicate that residual infestation, albeit geographically widespread, tended to be more common in the southern municipalities of the Department, as well as in Cercado and in some south-western municipalities – that is, in areas where baseline risk was also higher. In addition, our eco-regional appraisal suggests that municipalities with a higher percentage of territory corresponding to montane dry forest had overall higher infestation rates than those dominated by either highland grasslands (the Andean Puna) or moist tropical forests (the Bolivian Yungas) (Table 2, Figure 1C,D). Moreover, only two out of 27 municipalities originally dominated by montane dry forest, Santiváñez and Tacachi, reported zero infestation – and they did so in just one year each, coinciding with small bug-search efforts (Tables S1 and S3).

Finally, the vast majority of the 7321 triatomines collected during entomological surveys carried out between 2007 and 2010 (the period for which data were available) were identified as *T. infestans*, with annual percentages typically ∼93–96%. In 2009, when only 428 vectors were collected, 73.1% of specimens were *T. infestans* (CI_95_ 68.7–77.1%) and 25.7% *T. sordida*; in the rest of years, *T. sordida* represented just ∼5% of catches, with fairly constant values suggesting little intervention effects on this latter species (see Table S2). Other species (*T. guasayana* and *P. megistus*) were very rare, with just 16 specimens collected over the four-year period assessed (Table S2). Therefore, infestation figures discussed in this paper refer primarily to *T. infestans*.

### Modeling

The linear mixed model in Table 3 suggests that, on average, a ∼28% (CI_95_ 6–44%) reduction of infestation odds was achieved across the study period for each 10-fold increase in control effort – represented by a fixed term measuring the (log_10_) amount of insecticide used per census inhabitant in each municipality during the previous year. In addition, average dwelling infestation rates correlated positively with rates ascertained the previous year (Table 3). The model also suggests that infestation odds rose by a factor of ∼3.5 (CI_95_ 1.64–7.30) for each 10-fold increase in the proportion of municipal territory originally corresponding to montane dry forests. The model estimates a strong negative effect of the HDI covariate (slope coefficient −2.16), but with a relatively large SE (1.03). This suggests that infestation odds were lower in municipalities with higher HDI (odds ratio [OR] 0.12), yet uncertainty about this estimate is substantial (CI_95_ 0.01 to 0.93). Table 3 also shows that the municipality random effect explained nearly 30% of the total variance (an estimate of intra-class correlation [42]) after controlling for the effects of covariates. Diagnostic plots showed no trends, with normally distributed residuals (details not shown).

**Table 3:** Dwelling infestation by triatomine bugs in 39 municipalities, Cochabamba, Bolivia, 2000–2011*: linear mixed model results, with intervention effort modeled as the amount of insecticide used per census inhabitant in the previous year (number of parameters *k* = 7; BIC = 881.63; AICc = 855.50).

	Estimate	SE	CI_95_	
Fixed effects: coefficient estimates			Lower	Upper
Intercept	−3.193	0.821	−4.834	−1.553
Control effort (previous year)	−0.322	0.134	−0.585	−0.058
Autocorrelation (time)	0.459	0.036	0.387	0.530
Dry forest	1.243	0.373	0.498	1.988
Human Development Index	−2.160	1.033	−4.249	−0.072

Random effects: REML variance component estimates	σ^2^	SE	%σ^2^	
Municipality	0.289	0.106	29.981	
Residual	0.674	0.056	70.019	
Ratio	0.429			

*No infestation surveys were conducted in 2002.

The model used *N* = 325 observations (year-specific municipality-level dwelling infestation rates), weighted by a measure of bug-search effort; the inclusion of one-year lagged covariates censored observations for year 2000 (see text for details).

BIC, Bayesian information criterion; AICc, second-order Akaike's information criterion.

All fixed-effect coefficient estimates were different from zero at the 5% level; SE, standard error; CI_95_, lower and upper limits of the 95% confidence interval.

REML, restricted maximum likelihood; σ^2^, random effect variance estimate; %σ^2^, percentage of total variance that is attributable to differences in average infestation among municipalities (‘Municipality’), also known as intra-class correlation, and to year-to-year variation in infestation rates within municipalities (‘Residual’). Ratio = Municipality/Residual variance estimates.

In our second model, the ‘intervention effort’ covariate was replaced by an ordinal ‘year’ predictor so that year-to-year changes in infestation could be quantified (Table 4). This model suggests that infestation odds decreased by nearly 90% in 2003 compared to 2001 (OR 0.11; CI_95_ 0.06–0.19) and by nearly 80% in 2004 compared to 2003 (OR 0.22; CI_95_ 0.14–0.34). Infestation remained largely stable afterwards, with all adjusted coefficients effectively indistinguishable from zero except for a moderate but significant decrease in 2006 compared to 2005 (OR 0.62; CI_95_ 0.43–0.89). Effect-size estimates for other covariates were similar to those derived from our first model, again suggesting temporal dependence of infestation and higher risk in municipalities within the montane dry forest eco-region (Tables 3 and 4). The slope coefficient estimate for the HDI covariate was again negative but even more imprecise than in the previous model, with the CI_95_ including zero. Finally, this model estimated intra-class correlation as 58.3% of the total variance (Table 4); again, diagnostic plots showed no obvious trends, albeit the distribution of residuals slightly departed from normality (details not shown). We note that, while more complex in structure, this second model had much lower AICc and BIC scores than the first, simpler specification (ΔAICc = 98.4, ΔBIC = 69.3; Tables 3 and 4), suggesting that the ‘year-ordinal’ covariate helps explain variation in infestation rates substantially better than the whole-period averaged effect of intervention effort.

**Table 4:** Dwelling infestation by triatomine bugs in 39 municipalities, Cochabamba, Bolivia, 2000–2011*: linear mixed model results, with year specified as an ordinal, fixed effect (number of parameters *k* = 15; BIC = 812.32; AICc = 757.11).

	Estimate	SE	CI_95_	
Fixed effects: coefficient estimates			Lower	Upper
Intercept	−1.545	0.922	−3.394	0.304
Year [2003 *vs*. 2001]	**−2.237**	0.295	−2.817	−1.657
Year [2004 *vs*. 2003]	**−1.525**	0.234	−1.986	−1.065
Year [2005 *vs*. 2004]	0.324	0.221	−0.110	0.759
Year [2006 *vs*. 2005]	**−0.478**	0.182	−0.836	−0.120
Year [2007 *vs*. 2006]	0.341	0.210	−0.072	0.753
Year [2008 *vs*. 2007]	−0.095	0.209	−0.506	0.315
Year [2009 *vs*. 2008]	−0.167	0.183	−0.527	0.193
Year [2010 *vs*. 2009]	0.183	0.182	−0.175	0.541
Year [2011 *vs*. 2010]	0.186	0.190	−0.189	0.560
Autocorrelation (time)	**0.140**	0.047	0.048	0.233
Dry forest	**1.401**	0.413	0.575	2.227
Human Development Index	−1.778	1.255	−4.330	0.774

Random effects: REML variance component estimates	σ^2^	SE	%σ^2^	
Municipality	0.587	0.177	58.251	
Residual	0.421	0.036	41.749	
Ratio	1.394			

*No infestation surveys were conducted in 2002.

The model used *N* = 325 observations (year-specific municipality-level dwelling infestation rates), weighted by a measure of bug-search effort; the inclusion of the one-year lagged temporal autocorrelation covariate censored observations for year 2000 (see text for details).

BIC, Bayesian information criterion; AICc, second-order Akaike's information criterion.

Fixed-effect coefficient estimates are in **bold** if different from zero at the 5% level; SE, standard error; CI_95_, lower and upper limits of the 95% confidence interval.

REML, restricted maximum likelihood; σ^2^, random effect variance estimate; %σ^2^, percentage of total variance that is attributable to differences in average infestation among municipalities (‘Municipality’), also known as intra-class correlation, and to year-to-year variation in infestation rates within municipalities (‘Residual’). Ratio = Municipality/Residual variance estimates.

## Discussion

We have presented a detailed appraisal of the effects of Chagas disease vector control in one of the most highly-endemic settings worldwide. We used linear mixed models in which the correlated structure of the data, with repeated municipality-level infestation rate measurements taken over time, was accounted for by including a municipality random effect. In addition, we controlled for the likely temporal dependence of infestation measured in consecutive years, as well as for potentially important ecological and socio-economic confounders. The results show impressive achievements: in a region historically scourged by hyperendemic Chagas disease, the success of the vector control program will in all likelihood translate into a better, healthier future for thousands. Yet, residual infestation foci were widespread and will require long-term action.

As for previous region-wide assessments of Chagas disease vector control interventions (e.g., [43]–[46]), our analyses have however several limitations. First, we use secondary data that may contain errors of different kinds. For example, the unusually low rate of dwelling infestation reported for Cercado in 2004 might have arisen from a data entry mistake; we checked this and other suspected errors with Cochabamba CDCP staff, who confirmed the data with their own records, but mistakes may have originated in the original data entry – i.e., before the data reached the central CDCP management unit. We nonetheless think that the signal-to-noise ratio is sufficiently high in the dataset to allow for valid inference. Second, we note that our approach of modeling year-to-year variation in infestation rates (Table 4) likely underestimates intervention effects, particularly for 2001–2004, because insecticide spraying was not perfectly synchronous across municipalities. We think, however, that this analysis provides an informative overview of how the campaign, as a whole, had a profound and sustained impact on domestic vector populations, while showing at the same time that residual infestation is an issue that will require specific policy and action. Our first model specification (Table 3) provides a more direct appraisal of intervention effects, but AICc and BIC scores suggest that it explains the data substantially worse than the second model (Table 4); this most likely reflects the fact that the whole-period averaged effect of intervention effort ignores temporal heterogeneity. Third, our dependent variable and covariates were municipality-level aggregates, which made it impossible to control for variation among dwellings within municipalities (e.g., as a result of household-level socio-economic status, housing characteristics, actual control interventions, or surrounding landscape); with our aggregate data, in addition, we could not assess the spatial distribution of residual/re-emerging infestation foci within municipalities. Such aggregate-level appraisals, known as ‘ecologic’ studies, are a major tool of epidemiological and social science research – and the only option when no individual-level data are available [47], [48]. However, assuming that aggregate-level effects apply at the individual level – the so-called ‘ecological fallacy’ – is clearly questionable [48], [49]. We therefore make no claims as to what determines infestation risk variation among individual dwellings, while noting at the same time that discounting the very strong correlation between CDCP activities and plummeting infestation rates would probably be nonsensical. The fact that the direction (sign) of coefficient estimates, both for focal predictors and for covariates, was fully consistent with biology-based expectations reinforces our confidence in the results of the models [50]. Based on the very large differences of AICc and BIC scores, we primarily focus on the results of our second model (Table 4) in the discussion that follows [40], [41].

There are several findings of our quantitative appraisal that, we believe, merit detailed consideration. First, the intervention had drastic immediate effects, with dwelling infestation odds plummeting by about 80–90% in each of the first two assessments (Table 4). The public health benefits of the campaign (and, indirectly, the returns of the investment it required) were therefore nearly immediately measurable, and this may be seen as a major argument for intervention advocacy in other settings. On the other hand, our analyses show no discernible changes in infestation rates after 2004, except for a moderate decrease in 2006 (Table 4); that no *increase* was detected over several years is an indication of the mid-term effectiveness of the program, but, at the same time, the absence of any measurable *decrease* emphasizes the persistence of residual infestation foci. Importantly, such residual/re-emerging foci are usually much smaller (i.e., with bugs at much lower densities), and hence more difficult to detect, than pre-spray foci; as a consequence, post-control infestation indices almost certainly underestimate the unobserved true rates [28], [51]–[53]. Risk estimates based on entomological survey data are, therefore, almost certainly biased downwards, particularly after 2004, and this may potentially hinder the rigorous planning and assessment of CDCP activities [28], [51]–[53]. Although routinely neglected, this bias can be substantial and should be taken into account in both vector ecology research and control program management [51]–[54].

Second, our results hint at the importance of accounting for the correlated structure of the data when analyzing longitudinal infestation records. Thus, net of other effects, municipality-level infestation rates measured in any given year were positively and significantly correlated with rates measured the previous year, clearly indicating temporal dependence (Tables 3 and 4). In addition, our mixed models estimated intra-class correlation at between ∼30% and ∼58% (Tables 3 and 4), suggesting that there were substantial differences in average infestation among municipalities and that such variation was not completely captured by model covariates; the higher percentage estimated by the second model indicates that much ‘residual’ variance (i.e., within-municipality temporal variation in infestation) was explained by the ‘year’ ordinal predictor (Table 4) [45].

Finally, our models allowed us to estimate (and adjust for) the effects of some major putative confounders. For example, we showed that eco-regional variation significantly modified dwelling infestation odds, which rose substantially as the percent of municipal territory originally covered by montane dry forests increased (Tables 3 and 4). This is likely related to the fact that wild *T. infestans* populations are preferentially associated with this eco-region in Cochabamba [16], [18], [31] and, as recently shown using molecular genetics, are highly connected with domestic/peridomestic populations [55]. The inclusion of the HDI covariate was intended to provide adjustment for coarse socio-economic differences between municipalities; both models estimated a negative effect of this covariate, but with large associated uncertainties (Tables 3 and 4). It is important to note, we believe, that ignoring covariate effects and variance components [37], [42] would not only result in the loss of valuable information: it would also yield overly precise and possibly misleading estimates of the effects of focal interest (see Figure S2).

Our analyses thus show that area-wide insecticide-spraying campaigns had drastic effects on dwelling infestation by triatomines, and suggest that this will readily translate into patent public health benefits: compared with a no-intervention scenario, potential contact between Chagas disease vectors and people was averted in about 150,000 dwellings in Cochabamba. Assuming a 20% overall human infection rate at baseline [7]–[9], [31], [32] and an average of four people per dwelling, nearly 500,000 susceptible people were protected; if we assume, in addition (and very conservatively), that incidence is between 600 and 900 new cases per 100,000 population and year in the absence of control measures [1], [5], [10], [23], [56], then we can very roughly estimate that about 2800–4300 new infections/year were averted by the CDCP since 2004. However, the observed (and almost certainly biased down [52], [53]) residual infestation in ∼3% of dwellings of at-risk municipalities indicates that about 230–350 new cases of infection by *T. cruzi* are still to be expected each year in Cochabamba.

Unpublished CDCP serological data suggest that the prevalence of infection among children <5 years (5 y) of age has remained stable at about 1.7% between 2006 and 2010 in the study area: overall annual values (Agresti-Coull CI_95_; number of samples) were 1.56% (1.25–1.93; *N* = 5274) in 2006; 1.63% (1.39–1.91; *N* = 9315) in 2007; 1.66% (1.17–2.34; *N* = 1931) in 2008; 2.63% (2.08–3.32; *N* = 2625) in 2009; and 1.60% (1.34–1.92; *N* = 7302) in 2010 (Kruskal-Wallis χ^2^ = 3.13, d.f. = 4, *p* = 0.54; data from municipalities with >90 serum samples tested in any given year; details not shown). With these data and the methods outlined in Box 1 of ref. [23], we can (again very coarsely) estimate incidence as ∼700 new infections per 100,000 children <5 y and year in the study area. Although admittedly very rough (and presented mainly for illustrative purposes), this estimate seems at odds with the “∼3% residual infestation” scenario described above; because congenital transmission alone cannot account for the observed prevalence or estimated incidence in this age group [57], and because no bug-detection method has 100% sensitivity [52], [53], we speculate that true post-control infestation values may be substantially higher than observed. A recent report from the rural Bolivian Chaco, where *T. infestans* rapidly re-infested treated houses, suggests that a vector-control campaign similar to the one we assessed had limited short-term impact on incidence, but uncertainty about force of infection estimates was substantial [4].

We emphasize, in any case, that current infection rates are ∼10 times lower than typically reported for Bolivian young children in the pre-CDCP era (e.g., 22.0% [5], 11.8% [8], or 24.0% [9]; see also refs. [31], [58]). This suggests that, at baseline, pediatric incidence was probably much higher (perhaps about 10 times higher) than our rough 2006–2010 estimate based on CDCP serological data – and therefore that overall annual incidence was indeed well above 600–900 cases/100,000. Even if methodological issues (survey sampling design, antibody detection techniques) and uncertainty about baseline figures likely contribute to the disparities, we think it safe to conclude that vector control activities probably account for most of these sharp, long-term declines in the prevalence and incidence of pediatric *T. cruzi* infection in Cochabamba [1], [5], [22], [23], [28], [59]–[61].

### Conclusions and outlook

Insecticide-based control of dwelling-infesting vector populations remains the core tool for primary Chagas disease prevention [1], [5], [10], [28], [62]. The impressive achievements of the coordinated, international Initiative undertaken in the early 1990s across the Southern Cone countries of South America firmly established this view as a major public health dogma [1], [5], [10], [27], [56], [61]. This success was later replicated with the effective control, and likely elimination, of accidentally-introduced *Rhodnius prolixus* populations from Central America and southern Mexico [24], [25], [44]–[46]. However, and ironically, some of the most problematic territories, where the disease is highly endemic and its principal vector, *T. infestans*, is a widespread native pest, did not implement large-scale control programs until the late 1990s. This was the case of the Department of Cochabamba. Unfortunately, no systematic control measures are currently in place in some areas of the Gran Chaco where *T. infestans* is also the main vector [22], [62]. In parts of Mexico, Colombia, Venezuela, Ecuador, or Peru, important vector species such as *T. infestans*, *T. dimidiata* or *R. prolixus* are still commonly found infesting dwellings [62]. Highly coordinated vector control campaigns such as those described here and elsewhere (e.g., [23]–[25], [43]–[46]) are urgently needed in all these countries and territories.

Our appraisal demonstrates that ‘classical’ area-wide vector control campaigns have a crucial role to play in the endemic settings where resource-limited communities endure the highest risk of Chagas disease. Yet, by showing that residual dwelling infestation is relatively common despite intensive and highly effective control efforts, our findings also underscore the need for fully operational, long-term entomological-epidemiological surveillance systems [28]. This will require judicious, far-reaching public health policies capable of galvanizing sustained (and sustainable) preventive action [10], [22], [28], [62]. In Cochabamba, the relatively high rates of residual infestation in the municipality of Cercado, which includes the densely populated capital city, are particularly worrying; determining the relative importance of control failures (e.g., due to operational constraints or insecticide resistance) and true re-infestation of successfully-treated dwellings by wild vectors should be given high priority.

## Supporting Information

Figure S1 Observed proportions of dwellings infested by triatomine bugs in at-risk municipalities, Cochabamba, Bolivia, 2000–2011. Each municipality is represented by one color, with year-specific data linked by a line. Note the sharp decline of infestation rates and the persistence of residual infestation, with higher rates (particularly from 2005 to 2010) in one municipality, which corresponds to Cercado. No data were available for 2002.
(TIFF)

Figure S2 95% confidence intervals (CIs) of year-to-year infestation odds ratios (ORs) calculated with standard 2×2 contingency-table analyses (unadjusted) and estimated from the model in Table 4 of the main text (adjusted). Note the extreme underestimation of uncertainty in unadjusted ORs, which have unreliably small CIs, and how this leads to likely spurious “statistically significant” results at the 5% level (unadjusted CIs not crossing the grey dotted line at OR = 1 but adjusted CIs doing so) in four out of nine comparisons; note also the apparent overestimation of the effect in the 2004 *vs.* 2003 comparison. For graphic clarity, OR estimates are not presented; in the log_10_ scale of the *y*-axis, they are located at the center of each CI. Grey/white bands highlight CIs derived from the same year-to-year comparison (as indicated on the *x*-axis).
(TIFF)

Table S1 Proportion of dwellings searched for triatomine bugs (P(searched)) during the activities of the Chagas Disease Control Program of the Department of Cochabamba, Bolivia, 2000–2011 (no activities were conducted in 2002), and dwelling infestation rates (DIR) in each municipality and year.
(PDF)

Table S2 Triatomine bugs collected during the activities of the Chagas Disease Control Program of the Department of Cochabamba, Bolivia, 2007–2010.
(PDF)

Table S3 Municipalities with recorded dwelling infestation by triatomine bugs equal to zero in Chagas disease risk areas, Cochabamba, Bolivia, 2000–2011.
(PDF)
