# Supplementary figures and images for: Chagas Disease Vector Control in a Hyperendemic Setting: The First 11 Years of Intervention in Cochabamba, Bolivia
Source: PLoS Negl Trop Dis. 2014 Apr 3;8(4):e2782. doi: 10.1371/journal.pntd.0002782 (PMC3974664; doi:10.1371/journal.pntd.0002782)

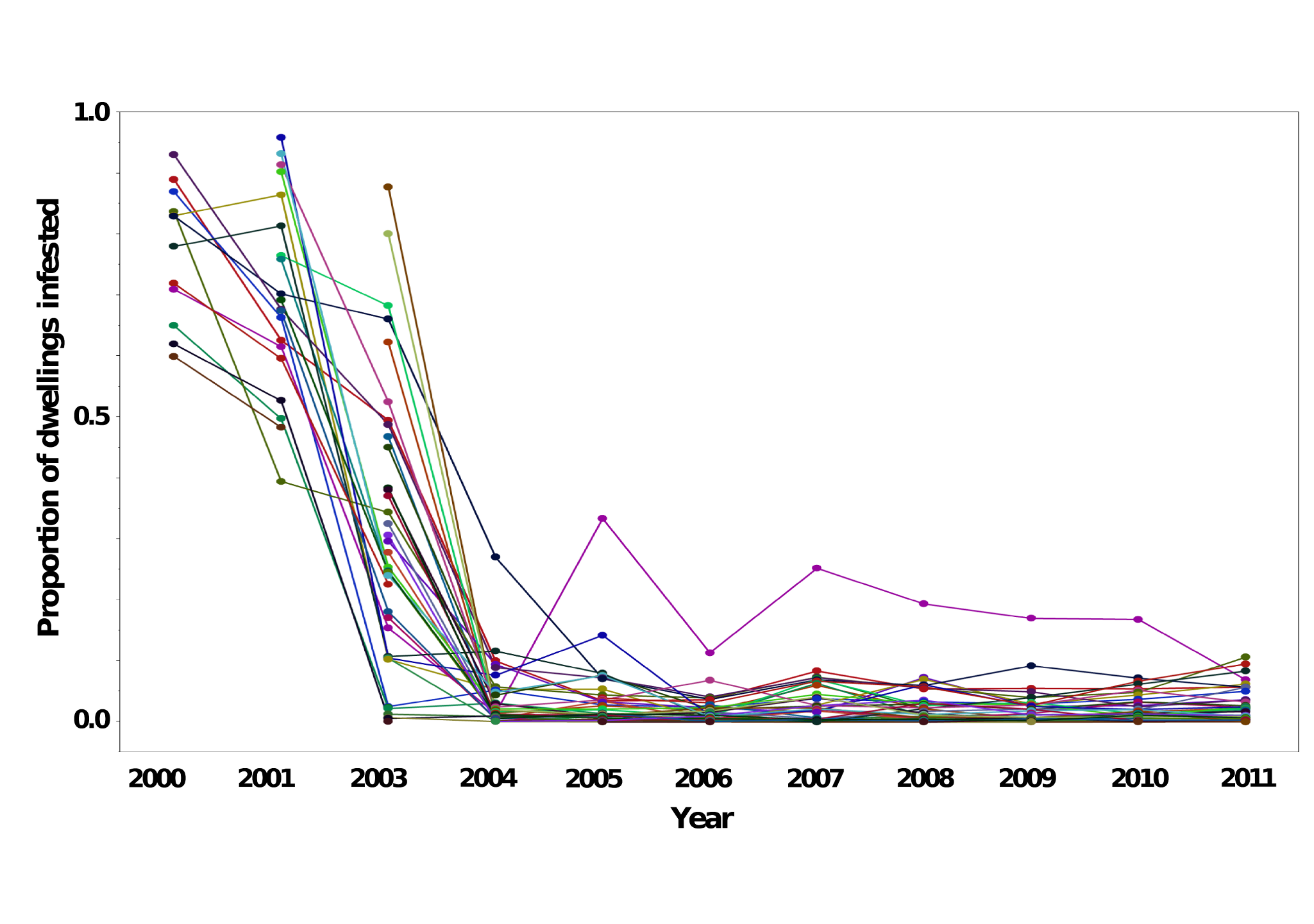

Supplement: Figure S1 — Observed proportions of dwellings infested by triatomine bugs in at-risk municipalities, Cochabamba, Bolivia, 2000–2011. Each municipality is represented by one color, with year-specific data linked by a line. Note the sharp decline of infestation rates and the persistence of residual infestation, with higher rates (particularly from 2005 to 2010) in one municipality, which corresponds to Cercado. No data were available for 2002. (TIFF) [file pntd.0002782.s001.tiff]

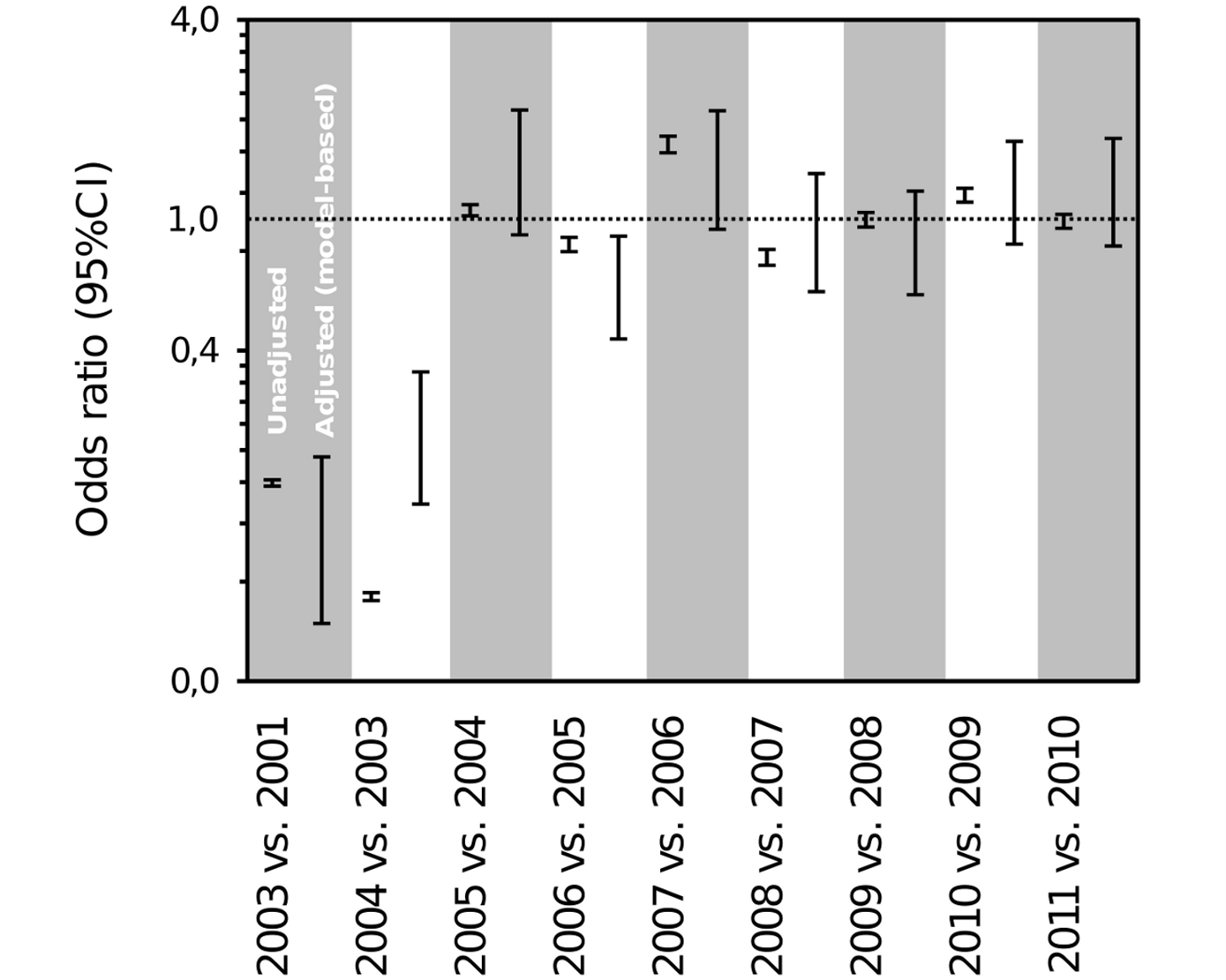

Supplement: Figure S2 — 95% confidence intervals (CIs) of year-to-year infestation odds ratios (ORs) calculated with standard 2×2 contingency-table analyses (unadjusted) and estimated from the model in Table 4 of the main text (adjusted). Note the extreme underestimation of uncertainty in unadjusted ORs, which have unreliably small CIs, and how this leads to likely spurious “statistically significant” results at the 5% level (unadjusted CIs not crossing the grey dotted line at OR = 1 but adjusted CIs doing so) in four out of nine comparisons; note also the apparent overestimation of the effect in the 2004 vs. 2003 comparison. For graphic clarity, OR estimates are not presented; in the log10 scale of the y-axis, they are located at the center of each CI. Grey/white bands highlight CIs derived from the same year-to-year comparison (as indicated on the x-axis). (TIFF) [file pntd.0002782.s002.tiff]
