# Supplementary material for: Chagas Disease Vector Control in a Hyperendemic Setting: The First 11 Years of Intervention in Cochabamba, Bolivia
Source: PLoS Negl Trop Dis. 2014 Apr 3;8(4):e2782. doi: 10.1371/journal.pntd.0002782 (PMC3974664; doi:10.1371/journal.pntd.0002782)
Supplement: Table S1 — Proportion of dwellings searched for triatomine bugs (P(searched)) during the activities of the Chagas Disease Control Program of the Department of Cochabamba, Bolivia, 2000–2011 (no activities were conducted in 2002), and dwelling infestation rates (DIR) in each municipality and year. (PDF) [file pntd.0002782.s003.pdf]

Table S1. Proportion of dwellings searched for triatomine bugs (P(searched)) during the activities of the Chagas Disease Control Program of the Department of Cochabamba, Bolivia, 2000–2011 (no activities were conducted in 2002), and dwelling infestation rates (DIR) in each municipality and year

Municipality	Year	Dwellings*	P(searched)	DIR
Aiquile	2000	6734	0.29	0.88992
Aiquile	2001	6870	0.89	0.62650
Aiquile	2003	7137	1.00	0.49501
Aiquile	2004	7265	1.00	0.10034
Aiquile	2005	7389	1.00	0.03490
Aiquile	2006	7510	1.00	0.03673
Aiquile	2007	7627	0.61	0.08358
Aiquile	2008	7739	0.93	0.05409
Aiquile	2009	7846	0.98	0.05516
Aiquile	2010	7948	0.84	0.05439
Aiquile	2011	8099	0.45	0.05743
Alalay	2000	1252	0.00	
Alalay	2001	1288	0.00	
Alalay	2003	1359	1.00	0.02353
Alalay	2004	1394	0.00	
Alalay	2005	1430	0.22	0.00000
Alalay	2006	1288	0.00	
Alalay	2007	1499	0.05	0.07143
Alalay	2008	1535	0.69	0.02174
Alalay	2009	1569	0.94	0.00542
Alalay	2010	1602	0.57	0.00769
Alalay	2011	1602	0.00	
Anzaldo	2000	2431	1.00	0.86983
Anzaldo	2001	2400	0.70	0.66369
Anzaldo	2003	2334	1.00	0.02545
Anzaldo	2004	2297	1.00	0.05242
Anzaldo	2005	2266	1.00	0.02809
Anzaldo	2006	2235	1.00	0.02411
Anzaldo	2007	2204	0.98	0.03841
Anzaldo	2008	2172	0.90	0.03327
Anzaldo	2009	2141	1.00	0.02954
Anzaldo	2010	2110	1.00	0.03750
Anzaldo	2011	2154	1.00	0.05046
Arani	2000	3104	0.00	
Arani	2001	3040	0.00	
Arani	2003	2906	1.00	0.62290
Arani	2004	2837	1.00	0.01437

Arani	2005	2767	1.00	0.02645
Arani	2006	2696	1.00	0.01887
Arani	2007	2623	1.00	0.06009
Arani	2008	2550	1.00	0.02126
Arani	2009	2476	1.00	0.00592
Arani	2010	2402	1.00	0.01734
Arani	2011	2451	1.00	0.02228
Arbieto	2000	2443	0.70	0.65029
Arbieto	2001	2477	0.49	0.49751
Arbieto	2003	2540	1.00	0.02210
Arbieto	2004	2567	1.00	0.03101
Arbieto	2005	2594	0.94	0.01391
Arbieto	2006	2615	0.73	0.01102
Arbieto	2007	2636	0.18	0.06342
Arbieto	2008	2662	1.00	0.00996
Arbieto	2009	2683	1.00	0.00371
Arbieto	2010	2701	1.00	0.00836
Arbieto	2011	2757	1.00	0.02380
Arque	2000	2942	0.00	
Arque	2001	3005	0.00	
Arque	2003	3129	0.68	0.29675
Arque	2004	3189	0.20	0.09455
Arque	2005	3248	0.43	0.03314
Arque	2006	3306	0.50	0.02340
Arque	2007	3363	0.26	0.01617
Arque	2008	3418	0.30	0.07303
Arque	2009	3471	0.44	0.02566
Arque	2010	3523	0.60	0.02685
Arque	2011	3590	0.36	0.03689
Capinota	2000	4422	0.07	0.83077
Capinota	2001	4427	0.85	0.86496
Capinota	2003	4446	1.00	0.10278
Capinota	2004	4459	1.00	0.05340
Capinota	2005	4471	1.00	0.05386
Capinota	2006	4482	0.48	0.00469
Capinota	2007	4496	0.30	0.02778
Capinota	2008	4506	0.40	0.07017
Capinota	2009	4517	0.92	0.02954
Capinota	2010	4527	0.65	0.04453
Capinota	2011	4617	0.58	0.06222
Cliza	2000	5193	0.00	
Cliza	2001	5238	0.85	0.75905
Cliza	2003	5312	0.24	0.24750
Cliza	2004	5345	1.00	0.00929
Cliza	2005	5371	0.87	0.00725

Cliza	2006	5394	0.68	0.00245
Cliza	2007	5410	0.29	0.00567
Cliza	2008	5422	0.75	0.00984
Cliza	2009	5431	0.71	0.01225
Cliza	2010	5434	0.77	0.01357
Cliza	2011	5549	0.80	0.01107
Cochabamba	2000	132727	0.10	0.70999
Cochabamba	2001	135195	0.01	0.61593
Cochabamba	2003	140009	0.59	0.15432
Cochabamba	2004	142319	0.65	0.01448
Cochabamba	2005	144555	0.01	0.33402
Cochabamba	2006	146714	0.02	0.11371
Cochabamba	2007	148814	0.01	0.25224
Cochabamba	2008	150836	0.01	0.19370
Cochabamba	2009	152767	0.03	0.16999
Cochabamba	2010	154596	0.04	0.16828
Cochabamba	2011	157826	0.08	0.06950
Colcapirhua	2000	10247	0.00	
Colcapirhua	2001	10894	0.00	
Colcapirhua	2003	12267	0.53	0.24539
Colcapirhua	2004	12993	0.34	0.00861
Colcapirhua	2005	13741	0.64	0.00954
Colcapirhua	2006	14512	0.28	0.00771
Colcapirhua	2007	15306	0.16	0.00417
Colcapirhua	2008	16120	0.23	0.00924
Colcapirhua	2009	16953	0.16	0.00789
Colcapirhua	2010	17801	0.13	0.00625
Colcapirhua	2011	18159	0.17	0.00612
Cuchumuela	2000	475	0.00	
Cuchumuela	2001	475	0.00	
Cuchumuela	2003	471	0.90	0.46809
Cuchumuela	2004	470	1.00	0.00586
Cuchumuela	2005	467	1.00	0.00187
Cuchumuela	2006	464	1.00	0.02800
Cuchumuela	2007	461	1.00	0.00702
Cuchumuela	2008	457	1.00	0.00174
Cuchumuela	2009	452	1.00	0.00333
Cuchumuela	2010	446	1.00	0.00677
Cuchumuela	2011	455	1.00	0.01903
Independencia	2000	7151	0.00	
Independencia	2001	7055	0.00	
Independencia	2003	6847	0.85	0.17128
Independencia	2004	6735	0.43	0.00553
Independencia	2005	6617	0.48	0.00221
Independencia	2006	6495	0.29	0.00539

Independencia	2007	6369	0.24	0.02057
Independencia	2008	6239	0.37	0.00613
Independencia	2009	6105	0.38	0.01427
Independencia	2010	5977	0.29	0.03260
Independencia	2011	6090	0.47	0.02406
Mizque	2000	6795	0.47	0.83749
Mizque	2001	6963	0.83	0.39466
Mizque	2003	7296	1.00	0.34399
Mizque	2004	7460	1.00	0.05764
Mizque	2005	7620	1.00	0.04447
Mizque	2006	7779	1.00	0.03973
Mizque	2007	7936	0.55	0.07348
Mizque	2008	8088	1.00	0.05576
Mizque	2009	8236	1.00	0.04032
Mizque	2010	8381	1.00	0.04876
Mizque	2011	8540	0.44	0.10640
Morochata	2000	8722	0.00	
Morochata	2001	8919	0.00	
Morochata	2003	9301	0.39	0.10481
Morochata	2004	9484	0.15	0.00069
Morochata	2005	9659	0.30	0.00905
Morochata	2006	9827	0.10	0.01581
Morochata	2007	9989	0.05	0.00000
Morochata	2008	10142	0.14	0.00287
Morochata	2009	10288	0.13	0.00686
Morochata	2010	10424	0.07	0.00958
Morochata	2011	10623	0.01	0.02105
Omereque	2000	1562	0.48	0.83019
Omereque	2001	1588	0.61	0.70237
Omereque	2003	1639	1.00	0.66111
Omereque	2004	1663	0.70	0.27062
Omereque	2005	1686	1.00	0.07109
Omereque	2006	1709	1.00	0.03723
Omereque	2007	1730	1.00	0.06938
Omereque	2008	1749	1.00	0.06001
Omereque	2009	1767	1.00	0.09232
Omereque	2010	1786	1.00	0.07203
Omereque	2011	1820	0.93	0.05716
Pasorapa	2000	1235	0.65	0.93052
Pasorapa	2001	1224	0.45	0.67748
Pasorapa	2003	1200	1.00	0.48797
Pasorapa	2004	1185	1.00	0.08941
Pasorapa	2005	1172	1.00	0.07245
Pasorapa	2006	1156	1.00	0.04134
Pasorapa	2007	1140	0.63	0.07293

Pasorapa	2008	1125	1.00	0.05547
Pasorapa	2009	1107	1.00	0.04961
Pasorapa	2010	1090	1.00	0.02492
Pasorapa	2011	1110	1.00	0.03594
Pocona	2000	3552	0.00	
Pocona	2001	3541	0.00	
Pocona	2003	3503	0.84	0.27832
Pocona	2004	3476	0.48	0.00594
Pocona	2005	3446	0.88	0.03413
Pocona	2006	3409	0.82	0.00430
Pocona	2007	3368	0.24	0.01820
Pocona	2008	3324	0.83	0.00470
Pocona	2009	3276	0.72	0.00212
Pocona	2010	3223	0.70	0.00176
Pocona	2011	3284	0.66	0.00414
Pojo	2000	8510	0.00	
Pojo	2001	9070	0.00	
Pojo	2003	10263	0.33	0.45041
Pojo	2004	10893	0.24	0.04405
Pojo	2005	11544	0.25	0.07721
Pojo	2006	12216	0.34	0.02104
Pojo	2007	12910	0.36	0.02615
Pojo	2008	13625	0.31	0.02931
Pojo	2009	14359	0.27	0.02077
Pojo	2010	15112	0.24	0.03305
Pojo	2011	5262	0.38	0.02672
Punata	2000	6945	0.00	
Punata	2001	6871	0.85	0.76550
Punata	2003	6708	0.38	0.68300
Punata	2004	6617	1.00	0.01682
Punata	2005	6519	1.00	0.02026
Punata	2006	6414	0.54	0.00921
Punata	2007	6303	0.08	0.07010
Punata	2008	6187	0.51	0.02701
Punata	2009	6062	0.77	0.03195
Punata	2010	5931	0.98	0.01357
Punata	2011	6056	0.34	0.02196
Quillacollo	2000	26169	0.00	
Quillacollo	2001	27163	0.00	
Quillacollo	2003	29138	0.63	0.32539
Quillacollo	2004	30121	0.59	0.00693
Quillacollo	2005	31094	0.69	0.00961
Quillacollo	2006	32050	0.21	0.01669
Quillacollo	2007	32991	0.15	0.00577
Quillacollo	2008	33916	0.16	0.01761

Quillacollo	2009	34815	0.17	0.02154
Quillacollo	2010	35681	0.17	0.02015
Quillacollo	2011	36399	0.03	0.05709
Sacaba	2000	29001	0.00	
Sacaba	2001	30454	0.00	
Sacaba	2003	33477	0.63	0.38074
Sacaba	2004	35038	0.39	0.03005
Sacaba	2005	36627	0.84	0.00972
Sacaba	2006	38246	0.26	0.01613
Sacaba	2007	39893	0.08	0.03973
Sacaba	2008	41564	0.16	0.01440
Sacaba	2009	43255	0.13	0.00412
Sacaba	2010	44962	0.11	0.01447
Sacaba	2011	45847	0.10	0.01716
Sacabamba	2000	1219	1.00	0.59984
Sacabamba	2001	1236	0.91	0.48350
Sacabamba	2003	1274	0.00	
Sacabamba	2004	1296	1.00	0.00167
Sacabamba	2005	1313	1.00	0.00499
Sacabamba	2006	1331	1.00	0.00044
Sacabamba	2007	1347	1.00	0.00000
Sacabamba	2008	1356	1.00	0.00000
Sacabamba	2009	1369	1.00	0.00000
Sacabamba	2010	1385	1.00	0.00029
Sacabamba	2011	1414	1.00	0.00052
San Benito	2000	3344	0.00	
San Benito	2001	3338	0.83	0.90246
San Benito	2003	3318	0.48	0.25427
San Benito	2004	3303	1.00	0.02178
San Benito	2005	3284	1.00	0.02136
San Benito	2006	3260	0.62	0.02330
San Benito	2007	3233	0.28	0.04550
San Benito	2008	3202	0.62	0.03035
San Benito	2009	3166	0.89	0.02834
San Benito	2010	3126	0.69	0.00933
San Benito	2011	3192	0.87	0.02533
Santivañez	2000	1642	0.49	0.78027
Santivañez	2001	1646	0.84	0.81377
Santivañez	2003	1650	1.00	0.10742
Santivañez	2004	1652	0.97	0.11622
Santivañez	2005	1656	1.00	0.07965
Santivañez	2006	1659	0.94	0.00641
Santivañez	2007	1662	0.26	0.00000
Santivañez	2008	1667	1.00	0.02205
Santivañez	2009	1673	1.00	0.04029

Santivañez	2010	1679	1.00	0.06221
Santivañez	2011	1713	0.82	0.08345
Sicaya	2000	596	0.00	
Sicaya	2001	588	0.96	0.95907
Sicaya	2003	572	1.00	0.10507
Sicaya	2004	564	1.00	0.07651
Sicaya	2005	555	1.00	0.14231
Sicaya	2006	546	0.68	0.01081
Sicaya	2007	537	1.00	0.01869
Sicaya	2008	527	1.00	0.06117
Sicaya	2009	518	1.00	0.02552
Sicaya	2010	507	1.00	0.02067
Sicaya	2011	517	1.00	0.02483
Sipe Sipe	2000	7834	0.00	
Sipe Sipe	2001	8161	0.07	0.91400
Sipe Sipe	2003	8827	0.96	0.52513
Sipe Sipe	2004	9161	0.72	0.02443
Sipe Sipe	2005	9495	1.00	0.03647
Sipe Sipe	2006	9826	0.42	0.06859
Sipe Sipe	2007	10156	0.11	0.02703
Sipe Sipe	2008	10482	0.33	0.02339
Sipe Sipe	2009	10802	0.40	0.02785
Sipe Sipe	2010	11116	0.49	0.05218
Sipe Sipe	2011	11339	0.27	0.03279
Tacachi	2000	280	0.00	
Tacachi	2001	313	0.00	
Tacachi	2003	385	0.75	0.87805
Tacachi	2004	427	0.71	0.01980
Tacachi	2005	473	0.76	0.01105
Tacachi	2006	524	0.83	0.02299
Tacachi	2007	579	0.30	0.02299
Tacachi	2008	640	1.00	0.00737
Tacachi	2009	706	1.00	0.00732
Tacachi	2010	776	0.67	0.01734
Tacachi	2011	776	0.09	0.00000
Tacopaya	2000	3059	0.00	
Tacopaya	2001	3127	0.00	
Tacopaya	2003	3262	0.52	0.01303
Tacopaya	2004	3329	0.23	0.00935
Tacopaya	2005	3393	0.37	0.00079
Tacopaya	2006	3456	0.25	0.00114
Tacopaya	2007	3519	0.43	0.00265
Tacopaya	2008	3580	0.45	0.00615
Tacopaya	2009	3639	0.40	0.00204
Tacopaya	2010	3695	0.40	0.00341

Tacopaya	2011	3766	0.62	0.00388
Tapacari	2000	6588	0.00	
Tapacari	2001	6767	0.14	0.93254
Tapacari	2003	7127	0.29	0.24023
Tapacari	2004	7307	0.25	0.04855
Tapacari	2005	7484	0.29	0.07629
Tapacari	2006	7661	0.40	0.00912
Tapacari	2007	7836	0.38	0.02138
Tapacari	2008	8010	0.35	0.01247
Tapacari	2009	8182	0.45	0.01852
Tapacari	2010	8351	0.44	0.02049
Tapacari	2011	8513	0.37	0.00925
Tarata	2000	2253	0.84	0.62005
Tarata	2001	2255	0.51	0.52715
Tarata	2003	2259	0.75	0.00588
Tarata	2004	2261	1.00	0.01027
Tarata	2005	2263	1.00	0.01065
Tarata	2006	2266	0.89	0.00548
Tarata	2007	2269	0.94	0.00235
Tarata	2008	2271	1.00	0.00331
Tarata	2009	2273	0.89	0.00695
Tarata	2010	2276	1.00	0.00114
Tarata	2011	2324	1.00	0.00324
Tiquipaya	2000	8941	0.00	
Tiquipaya	2001	9882	0.00	
Tiquipaya	2003	12021	0.46	0.37057
Tiquipaya	2004	13224	0.46	0.01286
Tiquipaya	2005	14524	0.52	0.01143
Tiquipaya	2006	15935	0.33	0.00434
Tiquipaya	2007	17466	0.16	0.00501
Tiquipaya	2008	19124	0.09	0.03064
Tiquipaya	2009	20913	0.09	0.02107
Tiquipaya	2010	22830	0.13	0.00232
Tiquipaya	2011	23289	0.16	0.00293
Tiraque	2000	9116	0.00	
Tiraque	2001	9175	0.00	
Tiraque	2003	9278	0.54	0.00733
Tiraque	2004	9320	0.14	0.00075
Tiraque	2005	9355	0.23	0.00047
Tiraque	2006	9355	0.17	0.00000
Tiraque	2007	9384	0.00	
Tiraque	2008	9405	0.15	0.00000
Tiraque	2009	9420	0.08	0.00000
Tiraque	2010	9428	0.00	
Tiraque	2011	9429	0.00	

Toco	2000	1704	0.00	
Toco	2001	1697	1.00	0.69227
Toco	2003	1676	0.27	0.24615
Toco	2004	1665	1.00	0.01242
Toco	2005	1652	0.66	0.00644
Toco	2006	1636	0.93	0.00593
Toco	2007	1620	0.91	0.00272
Toco	2008	1603	1.00	0.00341
Toco	2009	1585	1.00	0.00466
Toco	2010	1564	1.00	0.01451
Toco	2011	1597	1.00	0.00666
Tolata	2000	1338	0.00	
Tolata	2001	1386	1.00	0.67455
Tolata	2003	1484	0.18	0.18113
Tolata	2004	1534	1.00	0.00486
Tolata	2005	1585	0.70	0.01444
Tolata	2006	1634	0.89	0.00068
Tolata	2007	1685	0.40	0.00445
Tolata	2008	1735	0.69	0.00167
Tolata	2009	1785	0.78	0.00503
Tolata	2010	1835	1.00	0.00306
Tolata	2011	1874	1.00	0.00321
Totora	2000	3470	0.00	
Totora	2001	3411	0.00	
Totora	2003	3282	1.00	0.30617
Totora	2004	3211	0.99	0.00094
Totora	2005	3139	1.00	0.00261
Totora	2006	3063	0.90	0.01019
Totora	2007	2987	0.59	0.02701
Totora	2008	2906	1.00	0.03607
Totora	2009	2824	1.00	0.01176
Totora	2010	2741	1.00	0.01364
Totora	2011	2793	1.00	0.01044
Vacas	2000	3127	0.00	
Vacas	2001	3195	0.00	
Vacas	2003	3334	1.00	0.00088
Vacas	2004	3409	0.00	
Vacas	2005	3486	0.09	0.00000
Vacas	2006	3563	0.10	0.00000
Vacas	2007	3642	0.00	
Vacas	2008	3722	0.10	0.00000
Vacas	2009	3801	0.09	0.00307
Vacas	2010	3801	0.00	
Vacas	2011	3882	0.00	
Vila Vila	2000	1198	0.63	0.71974

Vila Vila	2001	1203	0.73	0.59633
Vila Vila	2003	1212	1.00	0.22616
Vila Vila	2004	1216	0.00	
Vila Vila	2005	1216	1.00	0.03935
Vila Vila	2006	1218	1.00	0.03147
Vila Vila	2007	1218	0.59	0.06732
Vila Vila	2008	1217	1.00	0.05764
Vila Vila	2009	1217	1.00	0.02748
Vila Vila	2010	1213	1.00	0.06720
Vila Vila	2011	1236	0.70	0.09524
Villa Rivero	2000	1552	0.00	
Villa Rivero	2001	1540	0.00	
Villa Rivero	2003	1509	0.88	0.80120
Villa Rivero	2004	1493	1.00	0.02026
Villa Rivero	2005	1474	1.00	0.01297
Villa Rivero	2006	1454	1.00	0.01802
Villa Rivero	2007	1432	0.69	0.04125
Villa Rivero	2008	1409	1.00	0.01138
Villa Rivero	2009	1384	1.00	0.00789
Villa Rivero	2010	1357	1.00	0.00862
Villa Rivero	2011	1386	1.00	0.00962
Vinto	2000	7896	0.00	
Vinto	2001	8205	0.00	
Vinto	2003	8829	0.72	0.38390
Vinto	2004	9139	0.66	0.00581
Vinto	2005	9449	0.86	0.01352
Vinto	2006	9754	0.49	0.01139
Vinto	2007	10058	0.21	0.00386
Vinto	2008	10354	0.20	0.00444
Vinto	2009	10644	0.19	0.00195
Vinto	2010	10925	0.21	0.01135
Vinto	2011	11144	0.25	0.00722

*Estimated using population projections by the Bolivian Institute of Statistics (INE)
