# Supplementary material for: Chagas Disease Vector Control in a Hyperendemic Setting: The First 11 Years of Intervention in Cochabamba, Bolivia
Source: PLoS Negl Trop Dis. 2014 Apr 3;8(4):e2782. doi: 10.1371/journal.pntd.0002782 (PMC3974664; doi:10.1371/journal.pntd.0002782)
Supplement: Table S2 — Triatomine bugs collected during the activities of the Chagas Disease Control Program of the Department of Cochabamba, Bolivia, 2007–2010. (PDF) [file pntd.0002782.s004.pdf]

Table S2. Triatomine bugs collected during the activities of the Chagas Disease Control Program of the Department of Cochabamba, Bolivia, 2007–2010

A. By year

Year	Bugs captured	*T. infestans*	%	Agresti-Coull 95%CI		*T. sordida*	%	Others	%
				Lower	Upper				
2007	4027	3883	96.42	95.80	96.96	133	3.30	11	0.27
2008	1114	1054	94.61	93.12	95.80	60	5.39	0	0
2009	428	313	73.13	68.73	77.12	110	25.70	5	1.17
2010	1752	1633	93.21	91.93	94.30	119	6.79	0	0
**Total**	**7321**	**6883**	**94.02**	**93.45**	**94.54**	**422**	**5.76**	**16**	**0.22**

B. By municipality

Municipality	*T. infestans*	*T. sordida*	Others	Total	% *T. infestans*	% *T. sordida*
Aiquile	1722	134	9	1865	92.33	7.18
Anzaldo	35	6	1	42	83.33	14.29
Arani	93	9	0	102	91.18	8.82
Arque	36	14	1	51	70.59	27.45
Capinota	46	8	0	54	85.19	14.81
Cliza	53	0	0	53	100	0
Cochabamba	1939	66	1	2006	96.66	3.29
Independencia	16	0	0	16	100	0
Mizque	1177	110	2	1289	91.31	8.53
Morochata	3	0	0	3	100	0
Omereque	414	37	1	452	91.59	8.19
Pasorapa	316	13	1	330	95.76	3.94
Pocona	2	0	0	2	100	0
Pojo	313	9	0	322	97.20	2.80
Punata	161	0	0	161	100	0
Quillacollo	41	0	0	41	100	0
Sacaba	122	1	0	123	99.19	0.81
San Benito	1	0	0	1	100	0
Santivañez	37	0	0	37	100	0
Sicaya	56	7	0	63	88.89	11.11
Sipe Sipe	34	0	0	34	100	0
Tacopaya	2	0	0	2	100	0
Tapacarí	109	0	0	109	100	0
Tiquipaya	4	8	0	12	33.33	66.67
Totora	74	0	0	74	100	0
Vila Vila	77	0	0	77	100	0
**Total**	**6883**	**422**	**16**	**7321**	**94.02**	**5.76**

C. Natural infection with *Trypanosoma cruzi*

Year	Bugs examined	Infected	% infected
2007	1017	44	4.33
2008	654	81	12.39
2009	359	35	9.75
2010	1109	88	7.94
**Total**	**3139**	**248**	**7.90**
