# Supplementary material for: Chagas Disease Vector Control in a Hyperendemic Setting: The First 11 Years of Intervention in Cochabamba, Bolivia
Source: PLoS Negl Trop Dis. 2014 Apr 3;8(4):e2782. doi: 10.1371/journal.pntd.0002782 (PMC3974664; doi:10.1371/journal.pntd.0002782)
Supplement: Table S3 — Municipalities with recorded dwelling infestation by triatomine bugs equal to zero in Chagas disease risk areas, Cochabamba, Bolivia, 2000–2011. (PDF) [file pntd.0002782.s005.pdf]

Table S3. Municipalities with recorded dwelling infestation by triatomine bugs equal to zero in Chagas disease risk areas, Cochabamba, Bolivia, 2000–2011*

Eco-region●	Municipality	Year	Dwellings#
Dry forest	Santiváñez	2007	433
	Tacachi	2011	73
Puna	Alalay	2005	311
	Sacabamba	2007	1664
		2008	2958
		2009	3094
	Vacas	2005	304
		2006	359
		2008	375
Yungas	Morochata	2007	490
	Tiraque	2006	1614
		2008	1423
		2009	759

*No data available for 2002
●A municipality is assigned to an eco-region when ≥50% of territory corresponds to that eco-region
#Number of dwellings surveyed each year (none was found infested)
